# Correlation of systemic arterial stiffness with changes in retinal and choroidal microvasculature in type 2 diabetes

**DOI:** 10.1038/s41598-018-37969-7

**Published:** 2019-02-04

**Authors:** Mirinae Kim, Rae-Young Kim, Joo-Young Kim, Young-Hoon Park

**Affiliations:** 1 Department of Ophthalmology and Visual Science, College of Medicine, The Catholic University of Korea, Seoul, Republic of Korea; 2 Catholic Institute for Visual Science, College of Medicine, The Catholic University of Korea, Seoul, Korea

## Abstract

This study was conducted to assess whether systemic arterial stiffness, indicated by cardio-ankle vascular index (CAVI), is related to changes in the microvasculature of the retina and choroid in diabetes mellitus (DM). This study included 113 patients with a confirmed diagnosis of type-2 DM. Among them, 18 patients did not have diabetic retinopathy (DR), 71 had non-proliferative DR (NPDR), and 24 had proliferative DR (PDR). The mean CAVI was 7.58 ± 1.41 in no DR, 8.72 ± 1.47 in NPDR, and 8.43 ± 1.25 in PDR group. Of the 113 eyes, 42 (37.2%) were classified as abnormal CAVI group (CAVI ≥ 9). This group had significantly higher cardiac autonomic neuropathy risk index score, decreased central choroidal thickness, and decreased choroidal vascularity index (CVI). Deep foveal avascular zone area was higher in the abnormal CAVI group. After adjustment for possible confounding factors, CAVI showed negative correlation with the CVI (*r* = −0.247, *P* = 0.013). In conclusion, there was a significant correlation between arteriosclerosis and choroidal vascular changes in DR. We suggest prompt ophthalmic evaluation in patients with systemic arteriosclerosis. If the ophthalmologist notes advanced DR, the patient should be referred to a cardiovascular clinic for detailed evaluation of systemic arteriosclerosis.

## Introduction

Arterial stiffness is a term used to describe the degree of vascular rigidity due to reduced elasticity of the artery. Many studies have demonstrated the predictive value of arterial stiffness on systemic cardiovascular diseases^1–5^. The most important factor determining arterial stiffness is age; the elasticity of the arterial wall decreases with age and arterial stiffness increases. In addition, increased arterial stiffness can occur as a result of elevated blood pressure, smoking, obesity, and other systemic vascular diseases including diabetes mellitus (DM)^6^.

Arterial stiffness, termed arteriosclerosis, can be assessed noninvasively by measuring the pulse wave velocity (PWV)^1^, central blood pressure (CBP)^7^, or cardio-ankle vascular index (CAVI)^8^. Among these, CAVI is superior to PWV because it is not affected by blood pressure at the time of measurement^8,9^. CAVI increases with age. It is high in many arteriosclerotic diseases, such as cerebrovascular diseases, chronic kidney disease, carotid arteriosclerosis, and coronary artery disease; it is also related to many coronary risk factors including smoking, DM, hypertension, and dyslipidemia^8^.

Atherosclerosis is a narrowing of the arteries caused by a buildup of plaque, and the term is sometimes used as a specific subtype of arteriosclerosis. Atherosclerosis can be assessed by measuring the ankle-brachial index (ABI) or intima-media thickness of the common carotid artery. Studies on the association between arterial stiffness and atherosclerosis showed conflicting results^10,11^.

Recent advances in multimodal imaging have facilitated many studies on microvascular changes in diabetic retinopathy^12–15^. Further, attempts have been made to quantitatively analyze diabetic microvascular changes of the retina and choroid. In patients with DM, increased foveal avascular zone area^12^, decreased retinal perfusion density^12,14^, and decreased choroidal vascularity index (CVI)^15^ were associated with worsening diabetic retinopathy (DR). Moreover, in patients with DM, these microvascular changes were evident even without DR.

Generally, DR is a metabolic disorder caused by hyperglycemia, and it shares common risk factors with other diabetic microvascular complications including diabetic nephropathy and neuropathy. Some previous studies identified a relationship between diabetic retinopathy and systemic endothelial dysfunction with peripheral arterial stiffness^16–18^. However, these studies evaluated only the presence of DR^18^ or the grade of severity of DR^16,17^. To date, the association between arterial stiffness and diabetic ocular microvascular changes using quantitative vascular parameters has not been explored. Further, the underlying mechanism and the association between large-artery stiffness and diabetic ocular microvascular changes remain unclear.

In this study, we evaluated the CAVI and the quantitative vascular parameters of the retina and choroid in patients with type 2 DM. The aim of the current study was to assess whether systemic arterial stiffness, as indicated by CAVI, has an association with changes in the microvasculature of the retina and choroid in type 2 DM.

## Results

In total, 113 eyes of patients with diabetes were included in this study. We have summarized the demographic, ocular, and systemic characteristics of the subjects according to the severity of diabetic retinopathy in Table 1. Of the 113 eyes, 18 (15.9%) were classified as no DR, 71 (62.8%) as non-proliferative DR (NPDR), and 24 (21.2%) as proliferative diabetic retinopathy (PDR). There were no significant differences with regard to age, sex, blood pressure, visual acuity, intraocular pressure, or refractive error among the 3 groups. Disease duration (*P* < 0.001), glycated hemoglobin (HbA1c) (*P* < 0.001), and glycoalbumin level (*P* = 0.030) differed significantly between each DR group. The mean CAVI was 7.58 ± 1.41 in the no DR, 8.72 ± 1.47 in NPDR, and 8.43 ± 1.25 in the PDR group (*P* = 0.012). If the ABI was set into ordinal variables (as normal versus abnormal ABI), advanced DR severity and abnormal ABI showed a significant correlation (*P* = 0.042, linear-by-linear association test). However, the mean ABI was 1.06 ± 0.14 in the no DR, 1.05 ± 0.10 in NPDR, and 1.10 ± 0.10 in the PDR group; the differences were not statistically significant (*P* = 0.162).

**Table 1:** Demographic and Clinical Data According to Severity of Diabetic Retinopathy.

	No DR	NPDR	PDR	*P* value
Number of eyes	18	71	24	—
Age, years	55.94 ± 12.59	60.42 ± 7.75	56.96 ± 8.21	0.073
Sex, male (n)	7 (38.9%)	34 (47.9%)	5 (20.8%)	0.065
Current smoking (n)	8 (44.4%)	34 (47.9%)	9 (37.5%)	0.681
Years with diabetes	5.44 ± 3.97	13.63 ± 8.67	12.83 ± 6.66	<0.001
Systolic BP, mmHg	132.11 ± 11.08	135.55 ± 15.23	134.75 ± 14.37	0.668
Diastolic BP, mmHg	95.50 ± 10.44	83.25 ± 8.77	83.33 ± 7.51	0.863
Mean arterial BP, mmHg	100.37 ± 9.74	100.69 ± 9.75	100.47 ± 9.04	0.990
HbA1c, %	7.13 ± 1.13	7.86 ± 1.54	9.23 ± 2.48	<0.001
Fasting blood sugar, mg/dL	147.72 ± 43.11	153.85 ± 72.08	140.21 ± 34.93	0.640
eGFR, mL/min/1.73 m^2^	93.13 ± 17.79	76.86 ± 23.11	79.95 ± 29.08	0.038
Creatinine, mg/dL	0.77 ± 0.15	0.97 ± 0.45	0.90 ± 0.31	0.124
Glycoalbumin, %	18.48 ± 5.15	23.98 ± 8.32	31.27 ± 7.05	0.030
Total cholesterol, mg/dL	146.56 ± 38.02	163.80 ± 53.60	155.63 ± 42.73	0.385
HDL, mg/dL	48.56 ± 11.83	49.17 ± 12.55	50.08 ± 11.38	0.917
LDL, mg/dL	76.72 ± 30.21	79.87 ± 28.28	82.79 ± 26.16	0.787
TG, mg/dL	126.17 ± 48.88	133.37 ± 79.01	144.71 ± 68.98	0.231
BMI, kg/m^2^	27.87 ± 3.84	25.15 ± 3.47	25.10 ± 3.01	0.010
CAN risk index	51.60 ± 22.98	66.53 ± 16.18	74.52 ± 15.81	<0.001
Mean peripheral neuropathy index	0.89 ± 0.86	1.15 ± 1.21	1.81 ± 1.50	0.097
CAVI	7.58 ± 1.41	8.72 ± 1.47	8.43 ± 1.25	0.012
ABI	1.06 ± 0.14	1.05 ± 0.10	1.10 ± 0.10	0.162
Ophthalmic examinations				
BCVA (logMAR)	0.08 ± 0.11	0.10 ± 0.11	0.11 ± 0.16	0.657
IOP, mmHg	14.83 ± 2.77	15.08 ± 2.57	14.63 ± 2.68	0.746
SE	0.24 ± 1.85	−0.23 ± 1.80	−0.13 ± 1.10	0.291

Data are expressed as mean ± standard deviation (95% confidence interval).

ABI, ankle-brachial index; BCVA, best-corrected visual acuity; BMI, body mass index; BP, blood pressure; CAN, cardiac autonomic neuropathy; CAVI, cardio-ankle vascular index; DR, diabetic retinopathy; eGFR, estimated glomerular filtration rate; HbA1c, glycated hemoglobin; HDL, high-density lipoprotein; IOP, intraocular pressure; LDL, low-density lipoprotein; logMAR, logarithm of the minimum angle of resolution; NPDR, non-proliferative diabetic retinopathy; PDR, proliferative diabetic retinopathy; SE, spherical equivalent; TG, triglyceride.

Tables 2 and 3 display comparisons of demographic and clinical data according to CAVI and ABI values. Of the 113 eyes, 42 (37.2%) were classified as abnormal CAVI group (CAVI ≥ 9). cardiac autonomic neuropathy (CAN) risk index score was significantly higher in the abnormal CAVI group (62.54 ± 20.23 and 71.15 ± 14.33, for CAVI < 9 and CAVI ≥ 9, respectively, *P* = 0.018). We noted decreased central choroidal thickness (*P* = 0.002) and decreased CVI (*P* = 0.041) in the abnormal CAVI group. Deep foveal avascular zone (FAZ) area was higher in the abnormal CAVI group (0.62 ± 0.28 and 0.79 ± 0.36, for CAVI < 9 and CAVI ≥ 9, respectively, *P* = 0.005). However, retinal microvascular parameters including superficial FAZ area, superficial and deep FAZ circulatory index, and superficial and deep capillary vessel density showed no significant differences between the two CAVI groups (all *P* values > 0.05). Of the 113 eyes, 34 (30.1%) were classified as abnormally low or high ABI (ABI < 0.9 or ≥1.3). There were no significant differences in terms of ocular microvascular parameters between the two ABI groups (all *P* values > 0.05).

**Table 2:** Demographic and clinical data according to cardio-ankle vascular index (CAVI) value.

	CAVI < 9	CAVI ≥ 9	*P* value
Number of eyes	71	42	—
Age, years	56.08 ± 8.86	63.86 ± 6.58	<0.001
Current smoking (n)	38 (53.5%)	13 (31.0%)	0.018
Years with diabetes	10.46 ± 7.25	15.00 ± 9.11	0.004
HbA1c,%	7.78 ± 1.56	8.48 ± 2.20	0.061
eGFR, mL/min/1.73 m^2^	86.81 ± 22.31	68.78 ± 23.47	<0.001
Creatinine, mg/dL	0.85 ± 0.25	1.06 ± 0.54	0.017
BMI, kg/m^2^	26.15 ± 3.71	24.59 ± 3.06	0.023
CAN risk index	62.54 ± 20.23	71.15 ± 14.33	0.018
CAVI	7.63 ± 0.91	9.90 ± 1.05	—
ABI	1.06 ± 0.10	1.08 ± 0.10	0.082
Visual acuity (logMAR)	0.08 ± 0.12	0.13 ± 0.11	0.048
GC-IPL thickness, μm	114.57 ± 13.58	113.68 ± 14.64	0.744
RNFL thickness, μm	110.39 ± 18.39	106.90 ± 11.71	0.221
Central retinal thickness, μm	236.49 ± 24.87	228.36 ± 28.81	0.116
Central choroidal thickness, μm	233.65 ± 72.74	188.55 ± 65.95	0.002
Superficial FAZ area, mm^2^	0.42 ± 0.21	0.44 ± 0.12	0.431
Deep FAZ area, mm^2^	0.62 ± 0.28	0.79 ± 0.36	0.005
Superficial FAZ circulatory index	0.67 ± 0.11	0.67 ± 0.12	0.840
Deep FAZ circulatory index	0.66 ± 0.09	0.66 ± 0.08	0.655
Superficial capillary vessel density, %	24.51 ± 3.49	24.19 ± 3.58	0.417
Deep capillary vessel density, %	23.13 ± 4.31	24.50 ± 3.56	0.372
Choroidal vascularity index, %	35.11 ± 2.20	34.25 ± 2.36	0.041

Data are expressed as mean ± standard deviation (95% confidence interval).

ABI, ankle-brachial index; BMI, body mass index; CAN, cardiac autonomic neuropathy; CAVI, cardio-ankle vascular index; eGFR, estimated glomerular filtration rate; FAZ, foveal avascular zone; GC-IPL, ganglion cell-inner plexiform layer; logMAR, logarithm of the minimum angle of resolution; RNFL, retinal nerve fiber layer.

**Table 3:** Demographic and clinical data according to ankle-brachial index (ABI) value.

	Normal ABI (0.9–1.3)	Abnormally Low or High ABI (ABI <0.9 or ≥1.3)	*P* value
Number of eyes	79	34	—
Age, years	58.29 ± 8.24	60.56 ± 10.21	0.215
Current smoking (n)	33 (41.8%)	18 (52.9%)	0.278
Years with diabetes	11.56 ± 7.15	13.53 ± 10.36	0.317
HbA1c,%	7.99 ± 2.03	8.14 ± 1.18	0.690
eGFR, mL/min/1.73 m^2^	83.11 ± 25.14	68.56 ± 18.12	0.003
Creatinine, mg/dL	0.88 ± 0.40	1.04 ± 0.37	0.049
BMI, kg/m^2^	25.18 ± 3.69	26.48 ± 3.05	0.074
CAN risk index	65.71 ± 18.69	66.10 ± 18.71	0.820
CAVI	8.49 ± 1.14	8.43 ± 2.04	0.866
ABI	1.08 ± 0.06	1.02 ± 0.16	—
Visual acuity (logMAR)	0.10 ± 0.12	0.10 ± 0.99	0.947
GC-IPL thickness, μm	114.07 ± 14.14	114.64 ± 13.62	0.844
RNFL thickness, μm	109.54 ± 16.68	108.05 ± 15.43	0.655
Central retinal thickness, μm	234.26 ± 28.33	231.62 ± 22.22	0.629
Central choroidal thickness, μm	209.67 ± 71.89	233.65 ± 75.00	0.111
Superficial FAZ area, mm^2^	0.42 ± 0.19	0.44 ± 0.16	0.486
Deep FAZ area, mm^2^	0.68 ± 0.34	0.72 ± 0.28	0.560
Superficial FAZ circulatory index	0.66 ± 0.12	0.69 ± 0.09	0.195
Deep FAZ circulatory index	0.65 ± 0.09	0.69 ± 0.10	0.059
Superficial capillary vessel density, %	24.33 ± 3.34	24.55 ± 3.99	0.769
Deep capillary vessel density, %	23.38 ± 3.44	24.25 ± 4.86	0.220
Choroidal vascularity index, %	34.55 ± 2.45	34.20 ± 2.28	0.488

Data are expressed as mean ± standard deviation (95% confidence interval).

ABI, ankle-brachial index; BMI, body mass index; CAN, cardiac autonomic neuropathy; CAVI, cardio-ankle vascular index; eGFR, estimated glomerular filtration rate; FAZ, foveal avascular zone; GC-IPL, ganglion cell-inner plexiform layer; logMAR, logarithm of the minimum angle of resolution; RNFL, retinal nerve fiber layer.

**Figure 1:**
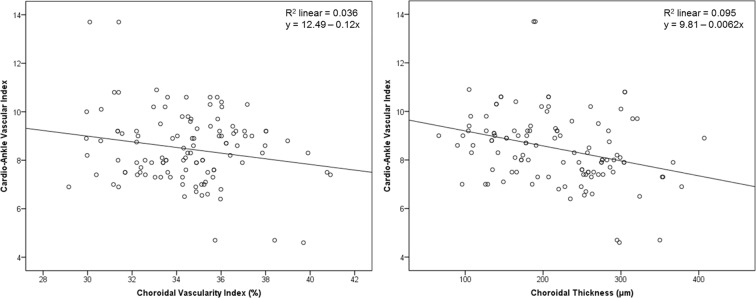
Pearson correlation between the cardio-ankle vascular index (CAVI) and the choroidal vascularity index (Left) and the choroidal thickness (Right). In this analysis, we adjusted for possible confounding factors including age, sex, smoking status, duration of diabetes, glycated hemoglobin, mean arterial blood pressure, fasting blood sugar, estimated glomerular filtration rate, creatinine, total cholesterol, body mass index, intraocular pressure, and spherical equivalent. CAVI was negatively correlated with choroidal vascularity index (*r* = −0.247, *P* = 0.013). However, CAVI exhibited no significant correlation with choroidal thickness (*P* = 0.115).

The results of Pearson’s correlation analysis are shown in Table 4 and Fig. 1. In this analysis, we adjusted for possible confounding factors including age, sex, smoking status, duration of diabetes, glycated hemoglobin, mean arterial blood pressure, fasting blood sugar, estimated glomerular filtration rate (eGFR), creatinine, total cholesterol, body mass index (BMI), intraocular pressure, and spherical equivalent. CAVI was negatively correlated with superficial capillary vessel density (*r* = −0.276, *P* = 0.045), and CVI (*r* = −0.247, *P* = 0.013). ABI exhibited no correlation with any ocular microvascular parameters as per Pearson’s correlation analysis (all *P* values > 0.05).

**Table 4:** Correlation analysis of cardio-ankle vascular index (CAVI) and retinal and choroidal vascular parameters.

Variables	Unadjusted		Adjusted*	
	*r*	*P* value	*r*	*P* value
GC-IPL thickness, μm	0.016	0.863	0.078	0.437
RNFL thickness, μm	−0.102	0.282	0.087	0.389
Central retinal thickness, μm	−0.089	0.347	−0.115	0.253
Central choroidal thickness, μm	−0.309	0.001	−0.125	0.115
Superficial FAZ area, mm^2^	0.084	0.376	0.201	0.054
Deep FAZ area, mm^2^	0.162	0.086	0.149	0.137
Superficial FAZ circulatory index	0.095	0.317	0.013	0.899
Deep FAZ circulatory index	−0.026	0.788	−0.004	0.966
Superficial capillary vessel density, %	−0.238	0.011	−0.276	0.045
Deep capillary vessel density, %	−0.070	0.459	−0.054	0.593
Choroidal vascularity index, %	−0.191	0.043	−0.247	0.013

*Adjusted for variables including age, sex, smoking status, duration of diabetes, glycated hemoglobin, mean arterial blood pressure, fasting blood sugar, eGFR, creatinine, total cholesterol, BMI, intraocular pressure, and spherical equivalent.

BMI, body mass index; CAVI, cardio-ankle vascular index; eGFR, estimated glomerular filtration rate; FAZ, foveal avascular zone; GC-IPL, ganglion cell-inner plexiform layer; logMAR, logarithm of the minimum angle of resolution; RNFL, retinal nerve fiber layer.

Among the 113 study participants, 6 (5.3%) patients showed asymmetric DR. The degrees of asymmetry were as follows; 3 patients had severe NPDR and PDR, 2 patients had moderate NPDR and severe NPDR, and 1 patient had moderate NPDR and PDR in each of the eyes. Three of these 6 patients were classified as abnormal CAVI group (CAVI ≥ 9). To validate our data, we conducted additional analysis only including 107 participants with symmetric DR; it showed the same results.

## Discussion

In this study, we aimed to assess whether systemic arterial stiffness was associated with the condition of the microvasculature of the retina and choroid in type 2 DM. Higher CAVI (which reflects stiffness of the aorta, femoral artery, and tibial artery) was associated with decreased CVI, and this correlation was statistically significant. CAVI could be used as a surrogate marker for DR progression, given that CVI exhibited some linear correlation with the progression of DR severity^15^. DR remains one of the most important diabetic microvascular complications and is a leading cause of irreversible blindness, highlighting the importance of early detection of progression and close monitoring.

There have been several reports of relationships between systemic arteriosclerosis and ocular diseases, such as branch retinal vein occlusion and age-related macular degeneration^19–21^. To the best of our knowledge, this is the first study to investigate the association between CAVI and microvascular changes in diabetes. Our findings revealed that systemic arterial stiffness, as measured by CAVI showed correlation ocular microvasculature changes in type 2 DM. The measurement protocol of CAVI is easy and the reproducibility of CAVI is also acceptable as per a previous report^22^. Furthermore, CAVI has the advantage that it is not affected by blood pressure (BP) at the time of measurement^8^. CAVI can be used as a physiologic surrogate marker for monitoring systemic arterial stiffness and the risk of DR progression in type 2 DM.

The underlying mechanisms of the association between arterial stiffness and ocular microvasculature changes are unclear. Our hypothesis is that, increased pulsatility due to the arterial stiffening affects the retinal and choroidal microvasculatures^23^. This increased pressure transmission to the microvessels may be the underlying pathophysiology of the increased risk of DR progression with increased CAVI. Another possible explanation is that, systemic arteriosclerosis and ocular microvasculature changes are both caused by the same underlying pathologic process of DM.

These associations were evident in choroidal vascular parameters rather than retinal vascular parameters. One possible mechanism of the lack of correlation between higher CAVI and disturbed retinal microvasculature may be due to the auto-regulation of retinal vessels. As is widely known, there is no neuronal innervation in the retinal vascular beds, and retinal blood flow is regulated by local mechanisms^24^. Due to the intrinsic autoregulatory mechanisms, retinal microvessels might be better protected from hypoxic damage compared to the choroidal vessels. Considering autoregulatory disturbances of retinal vessels in diabetic patients^25^, further studies are required with large-scale data with central retinal artery and vein equivalents, which could provide supportive evidence for our results.

In this study, arteriosclerosis was significantly correlated with ocular microvascular changes in diabetes, but atherosclerosis was not. Studies exploring the link between atherosclerosis, as measured by ABI, and DR have produced mixed results. In some reports, DR was associated with an abnormally low ABI (ABI < 0.9), but not with an abnormally high ABI (ABI ≥ 1.3)^26,27^. In contrast, in a recent study, abnormally low or high ABI was associated with PDR^28^. Previous studies evaluated only the grade of severity of DR. In our study population, the ABI did not show a correlation with the ocular microvascular parameters; quantitative analysis of the microvascular parameters in our study might provide stronger supportive evidence for our work.

Subjects with an excessively low ABI (<0.9) may have a severely arteriosclerotic femoral artery^22^. The data from these individuals should be cautiously analyzed while using CAVI due to the possibility of a false low CAVI score. In our study, 5 subjects with low ABI (<0.9) were included. The ABI of these 5 subjects ranged from 0.76 to 0.87, and the CAVI score ranged from 8.3 to 10.2. There was no statistical difference even after excluding these low ABI subjects (data not shown).

Our study had some limitations. First, systemic arteriosclerosis and DR might share common risk factors and both might be caused by the same underlying pathologic process of DM. This might cause underestimation of the associations between CAVI and ocular microvascular changes. And we did not collect data on the presence of carotid artery disease. Second, due to its retrospective design based on medical records, this study does not show changes of CAVI and ocular microvascular parameters over time, and if unrecognized variables, such as the use of statins or antihypertensive medications within each group, could have affected the results. Therefore, a longitudinal study is needed to confirm the pathophysiological mechanisms linking systemic arterial stiffness and ocular microvascular changes. Third, we did not evaluate the ocular blood flow. The effect of ocular blood flow regulation on DR remains unclear, and retinal capillary vessel density or CVI cannot accurately reflect the ocular blood flow. Fourth, FAZ area and retinal vessel density can be influenced by the spherical equivalent or axial length. We adjusted the spherical equivalent to exclude the confounding effect, but we did not analyze the axial length. Future studies should address these limitations.

In summary, systemic arteriosclerosis had a significant correlation with choroidal vascular changes in DR. Further longitudinal studies are necessary to support these findings and correlate them with structural and functional parameters. We suggest prompt ophthalmic evaluation in patients with systemic arteriosclerosis in DR. If the ophthalmologist notes advanced DR, the patient should be referred to a cardiovascular clinic for a detailed evaluation of systemic arteriosclerosis.

## Methods

### Study population

The study included patients with a confirmed diagnosis of type 2 DM. We recruited all participants between December 2016 and March 2018 at Seoul St. Mary’s Hospital in Korea and conducted a retrospective chart review. This retrospective study adhered to the tenets of the Declaration of Helsinki. Institutional Review Board (IRB)/Ethics Committee approval was obtained from the Catholic University of Korea, which waived the requirement for obtaining informed patient consent because of the retrospective nature of the study. In patients with symmetric DR, only the right eye was included in the analysis. In patients with asymmetric DR, the data of the worse eye was included.

Exclusion criteria for the study were as follows: (1) age over 75 years, (2) medication for dyslipidemia started prior to CAVI measurement, (3) diagnosis of cardiovascular disease besides DM, (4) presence of other retinal diseases, including glaucoma, age-related macular degeneration, epiretinal membrane, uveitis, or retinal vein occlusion, (5) clinically significant diabetic macular edema, and (6) eyes with a history of laser treatment, intravitreal injection, or intraocular surgery. Previous studies have demonstrated retinal and choroidal thinning even during the normal aging process^29,30^. To minimize the confounding effect of age-related retinal and choroidal thinning, we excluded patients aged over 75 years.

### General examination and laboratory tests

Patients were referred to the specialized endocrinology center, and a thorough evaluation for diabetes was performed. In the clinic, all patients were evaluated standardly for cardiovascular diseases (pulse-wave velocity using vascular screening system), peripheral neuropathy test (sensory nerve conduction threshold evaluation) and autonomic nervous system test. We retrospectively collected these medical records. We recorded the height, body weight, and BMI data. The systolic and diastolic BPs were measured at rest, and the mean arterial pressure was calculated as diastolic BP +1/3 (systolic BP − diastolic BP). Laboratory test data including glycated hemoglobin (HbA1c), fasting blood sugar, eGFR, creatinine, glycoalbumin and lipid profile [total cholesterol, high-density lipoprotein (HDL), low-density lipoprotein (LDL), and triglycerides (TG)] were obtained.

### Ophthalmic examination

All subjects underwent ocular examinations including best-corrected visual acuity (BCVA) evaluation (logarithm of the minimum angle of resolution [logMAR] scale), non-contact pneumatic tonometry, slit-lamp biomicroscopy, dilated fundus examination, and optical coherence tomography (OCT). OCT imaging was performed with a swept-source OCT device (DRI Triton, Topcon, Tokyo, Japan).

We defined and graded the severity of the DR according to the modified Early Treatment Diabetic Retinopathy Study (ETDRS) retinopathy severity scale^31^, and classified patients into 3 groups according to the DR severity as follows: no DR, NPDR, or PDR.

We obtained retinal and choroidal thicknesses using in-built OCT software. We created thickness maps in accordance with the standard ETDRS subfield, consisting of 3 concentric circular areas with 9 independent sectors, and used the central retinal and choroidal thicknesses of the innermost 1-mm circular area for analysis. The automated segmentation software of the OCT device can identify the outer boundary of the retinal nerve fiber layer (RNFL) and the inner plexiform layer (IPL). For every OCT scan, each segmented layer line can be manually adjusted to avoid possible segmentation errors. The difference between the RNFL and the IPL outer boundary segmentation yields the ganglion cell-IPL (GC-IPL) thickness. In this study, we measured the average macular GC-IPL thickness within an annulus with inner and outer diameters of 1-mm and 3-mm, respectively. We then used the average RNFL thickness at the peripapillary area for analysis.

**Figure 2:**
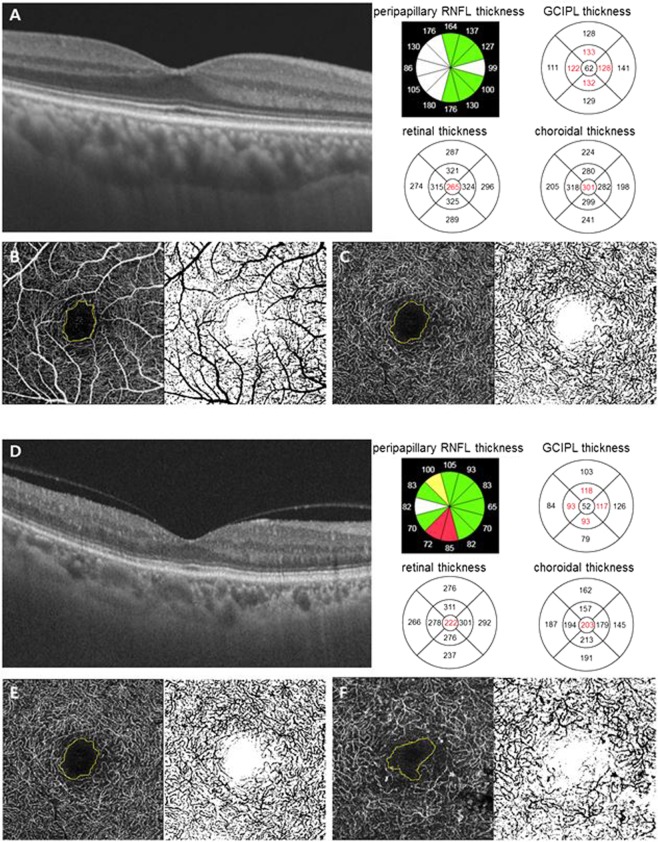
Representative optical coherence tomography (OCT) and OCT angiography images. Images from a patient with mild non-proliferative diabetic retinopathy (**A–C**) and from a patient with proliferative diabetic retinopathy (**D–F**). (**A,D**) We obtained retinal and choroidal thicknesses using in-built OCT software. Average RNFL thickness at peripapillary area, GC-IPL thickness within an annulus with inner and outer diameters of 1-mm and 3-mm, and central retinal and choroidal thicknesses of the innermost 1-mm circular area were used for analysis. (**B,C,E,F**) Representative 3 × 3 mm sized *en face* OCT angiography images demonstrate visualization of the superficial retinal capillary plexus and the deep retinal capillary plexus. We measured the FAZ area, FAZ circulatory index, and vessel density at the superficial and deep retinal capillary plexuses manually using ImageJ software. Notes: RNFL, retinal nerve fiber layer; GC-IPL, ganglion cell-inner plexiform layer.

We measured the FAZ area, FAZ circulatory index, and vessel density at the superficial and deep retinal capillary plexuses manually using ImageJ software (version 1.51; National Institutes of Health, Bethesda, MD, USA). The 3 × 3 mm sized *en-face* OCT angiography images at the superficial and deep capillary plexuses were used for analysis. Representative OCT images are shown in Fig. 2.

For quantitative analysis of the choroidal vasculature, we calculated the CVI in the total macular area. We used the segmentation protocol described by Agrawal *et al*.^32^. CVI, which represents the choroidal vascular component, was defined as the ratio of the vascular luminal area to the total choroidal area.

### Measurement of systemic arterial stiffness

We assessed arterial stiffness by measuring CAVI. Measurements of CAVI, ABI, and blood pressure were performed using the program embedded in the vascular screening system (VaSera VS-1000, Fukuda Denshi Co. Ltd, Tokyo, Japan). Electrocardiography (ECG) and phonocardiography (PCG) were performed, pressure and waveforms of the brachial and ankle arteries were evaluated, and brachial-ankle PWV (baPWV) and subsequently CAVI were calculated automatically^22,33^.

Ocular examination, measurement of CAVI, and the laboratory tests were performed within a 1-month time frame.

### Evaluation of cardiac autonomic neuropathy and peripheral neuropathy

We evaluated CAN using Ewing’s traditional five simple tests, namely, changes in the R-R with standing, deep breathing, and the Valsalva maneuver as well as changes in blood pressure in response to sustained handgrip and standing up^34^. Spectral analysis of heart rate variability (HRV) was performed, and the standard deviation of all normal R-R intervals (SDNN) and the root-mean square of the difference of successive R-R intervals (rMSSD) were evaluated. CAN risk index was assessed with a CAN analyzer (DiCAN, Medicore, Seoul, Korea). The CAN risk index value was classified as follows: normal <38.4; borderline 38.4–53.8; and abnormal >53.8.

We evaluated peripheral neuropathy by measuring the current perception threshold (CPT) using a neurometer (Neurotron Inc., Baltimore, MD, USA). The standard method of CPT testing has been described previously^35^. The neurometer generates a constant alternating current stimulus, which is applied to two different test sites, the median + ulnar nerves on the ring finger (C7, C8 level), and the deep and superficial peroneal nerves on toe 1 (L4, L5 level). We performed the data analysis using the Neuval^®^ software provided with the device, and used the mean CPT grade for analysis.

### Statistical analysis

We expressed categorical data as an absolute number, and continuous data as mean ± standard deviation (95% confidence interval). We performed statistical analysis using the Statistical Package for the Social Sciences for Windows version 23.0 (SPSS Inc., Chicago, IL, USA). The normality of data distribution was confirmed via the Kolmogorov–Smirnov test. Demographic and clinical data of patients were compared by one-way analysis of variance (ANOVA), chi-square, and Fisher’s exact tests. In cases of significant results (*P* < 0.05), the ANOVA was followed by a post-hoc Bonferroni correction. We used the linear-by-linear association test to examine the linear correlation between the advanced DR stage and abnormal ABI. We used Pearson’s correlation test to investigate the associations between CAVI and retinal and choroidal vascular parameters. Statistical significance was assumed if *P* < 0.05.

## Data Availability

The datasets during and/or analyzed during the current study are available from the corresponding author on reasonable request.
